# G**α**s Protein Expression Is an Independent Predictor of Recurrence in Prostate Cancer

**DOI:** 10.1155/2014/301376

**Published:** 2014-03-31

**Authors:** Lijuan Wang, Guihua Jin, Chenchen He, Xijing Guo, Xia Zhou, Meng Li, Xia Ying, Le Wang, Huili Wu, Qing Zhu

**Affiliations:** Department of Medical Oncology, The First Affiliated Hospital, Xi'an Jiao Tong University of Medical College, Xi'an 710061, China

## Abstract

*Background*. T393C polymorphism in the gene GNAS1, which encodes the G-protein alpha s subunit (G**α**s) of heterotrimeric G protein, is significantly associated with the clinical outcome of patients suffering from several cancers. However, studies on the role and protein expression of G**α**s subunit in prostate cancer were still unavailable. *Methods*. The immunohistochemical staining was used to assess G**α**s expression through tissue microarray procedure of 56 metastatic PCas, 291 localized PCas, and 67 benign hyperplasia (BPH). G**α**s expression was semiquantitatively scored and evaluated the correlation with pathologic parameters and biochemical recurrence of prostate-specific antigen (PSA). *Results*. G**α**s expression was localized in nuclear and cytoplasm in prostate cancer cells and downregulated in metastatic PCa compared to localized PCa and BPH (*P* < 0.001). G**α**s was inversely associated with PSA level and Gleason scores; patients with low expression of G**α**s had adverse clincopathological features. In multivariable Cox regression analysis, high G**α**s expression and Gleason scores were independent predictors of both PSA progression-free and overall survival. *Conclusions*. G**α**s down-expression is associated with adverse pathologic features and clinical PSA biochemical recurrence of prostate cancer. G**α**s is an independent predictor to help determine the risk of PSA progression and death.

## 1. Introduction

Prostate cancer (PCa), the most frequently diagnosed malignancy, has become the second leading cause of cancer-related deaths among men in Western countries [1, 2]. Endocrine therapies which aimed at inhibiting the androgen receptor (AR) function was the mainstay of treatment for advanced prostate cancer based on that the androgen signaling will promote the proliferation of prostate cancer cell. Unfortunately, most of treated patients progressed toward castration-resistant prostate cancer (CRPC) from castration-dependent prostate cancer. And CRPC characterized by aggressive growth and ability to colonize distal organs, which made CRPC still incurable and the median survival time for patients with CRPC was only 12 months [3]. The status of AR was highly predictive of prostate cancer patients that will benefit from endocrine therapy but was not correlated with a better clinical outcome [4, 5]. Prostate-specific antigen (PSA) is a protein produced by the prostate gland cells. The PSA test measures the level of PSA in a man's blood. Although many published studies have assessed the performance of candidate biomarkers in predicting time to relapse of prostate cancer following radical prostatectomy [6, 7], no molecular markers suitable for routine clinical practice that can identify those prostate cancer patients with a high risk of early clinical progression or prostate cancer-specific mortality have been found.

G-proteins are composed of *α*, *β*, and *γ* subunits and *α* subunit are classified into 4 families: G*α*s, G*α*i/o, G*α*q/11, and G*α*12/13. Each of them has multiple members with different expression specificity [8, 9]. Although G*α*s is the most extensively characterized and clinically relevant, literature is not unanimous on the role of G*α*s in different types of cancers. In lung cancer, Choi et al. found that G*α*s could augment cisplatin-induced apoptosis of lung cancer cells through upregulating Bak expression by increasing transcription and by decreasing the rate of protein degradation [10] and the efficacy of radiotherapy of lung cancer may be improved by modulating G*α*s signaling pathway [11]. But in cervical cancer or intrahepatic cholangiocarcinoma (ICC), the situation was just opposite. Cho et al. found G*α*s inhibited cisplatin-induced apoptosis by increasing transcription of X-linked inhibitor of apoptosis protein (XIAP) and by decreasing degradation of XIAP protein in HeLa cervical cancer cells [12]. In ICC, Schmitz et al. also found a significant association of both unfavorable disease-specific overall survival and recurrence-free survival with the homozygous TT genotype of the GNAS1 gene which encoded G*α*s protein [13, 14]. However, they also reported that T393C polymorphism in the gene GNAS1 was significantly associated with favorable clinical outcome of patients suffering from bladder cancer, chronic lymphocytic leukemia [15], and renal cell carcinoma [16].

The situation was even more complicated in prostate cancer. There were studies reported no association was found between GNAS T393T genotype and prostate cancer [17, 18]. But Liu et al. identified that membrane caveolae-associated Gas was involved in androgen receptor (AR) transactivation by modulating the activities of different PI3K isoforms [19]. More importantly, it had been reported that the expression of G*α*s and G*α*i decreased 30% to 40% after neoplastic transformation [20]. And the levels of G*α*s and G*α*i subunits correlated inversely with serum prostate specific antigen in patients with prostate cancer [20], which indicated an important regulatory role of G*α*s and G*α*i for cell proliferation and neoplastic transformation in human prostate cancer and they may have prognostic value. Therefore, more in-depth investigations are necessary to address this controversy and identify the role of G*α*s in prostate cancer.

Thus, we assessed the potential of G*α*s as a prognostic marker by determining the level of G*α*s protein expression in a series of 347 postradical prostatectomy prostate cancer tissue microarrays (TMA) which include 56 metastatic PCas and 291 localized PCas and 67 benign prostatic hyperplasia (BPH) as controls using immunohistochemistry (IHC). In the present study, we found that expression of G*α*s protein was decreased in high grade and metastatic PCas. And low G*α*s protein levels were strongly associated with adverse clinicopathologic features and poor clinical outcomes in metastatic and localized PCa patients. Multivariate Cox regression analysis showed that low expression of G*α*s was an independent predictor of prostate cancer recurrence and cancer-specific death in metastatic and localized PCa. To the best of our knowledge, this is the first study to identify the independent predictive role of G*α*s in patient with prostate cancer.

## 2. Patients and Methods

### 2.1. Patient Selection

In order to study G*α*s expression in prostate cancer by immunohistochemistry, a total of 347 formalin-fixed, paraffin-embedded prostate tissues between 1994 and 1997 were retrieved from the archives of the First Affiliated Hospital, College of Medicine, and Xi'an Jiao Tong University, and a tissue microarray (TMA) was constructed. The TMA included a series of 56 metastatic PCas and 291 localized PCas. In addition, 67 benign prostatic hyperplasia (BPH) samples were collected as control. This research project was approved by the Ethical Committee of the Xi'an Jiao Tong University, and all the patients had been given their fully written informed consent.

Data were collected on patients with disease baseline and clinicopathologic characteristics as well as 2 treatment outcomes: time to progression and prostate cancer-specific mortality (PCSM). Prostate cancers were graded based on the Gleason system by 2 independent pathologists at the First Affiliated Hospital, College of Medicine, and Xi'an Jiao Tong University in a blind and consecutive manner to ensure adequate diagnosis and grade. The TNM staging system was used to describe the extent of Prostate cancer in patients (based on the AJCC Cancer Staging Manual, Seventh Edition, 2010, Springer, New York, Inc.). TNM stages IIA and IIB were considered TNM stage II.

### 2.2. Immunohistochemistry (IHC)

Paraffin-embedded section of normal and tumor tissue was stained for G*α*s expression. Immunohistochemistry for G*α*s was performed as previous reported with slight modification. Briefly, slides were deparaffinized in xylene and rehydrated in a graded alcohol series before endogenous peroxidase activity was blocked with 3% H_2_O_2_ in methanol. After nonspecific protein binding was blocked, the primary antibody diluted into recommended concentration for G*α*s, which was purchased from Abcam (ab58810), was applied overnight in a humidity chamber at 4°C. Biotinylated secondary antibody was applied for 30 min at room temperature after washing with PBS for 3 times. Visualization was performed using DAB chromogen for 2 to 3 minutes. Negative control was conducted by replacing the primary antibody with preimmune rabbit serum.

### 2.3. Evaluation of Staining

**Figure 1:**
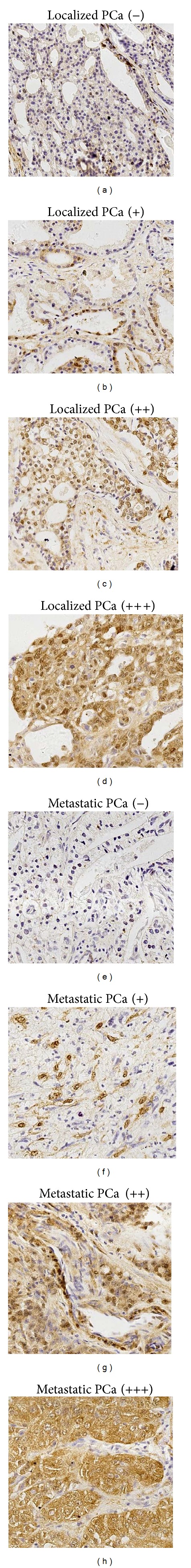
Immunohistochemically stained localized and metastatic PCa tissues from patients. (a) localized PCa tissues without G*α*s expression (−); (b) localized PCa tissues with weak G*α*s expression (+); (c) localized PCa tissues with moderate G*α*s expression (++); (d) localized PCa tissues with strong G*α*s expression (+++); (e) metastatic PCa tissues without G*α*s expression (−); (f) metastatic PCa tissues with weak G*α*s expression (+); (g) metastatic PCa tissues with moderate G*α*s expression (++); (h) metastatic PCa tissues with strong G*α*s expression (+++). Representative images were taken under a microscope (×20).

To evaluate G*α*s expression, we used the immunoreactive score (IRS) as previously implemented by Tischler et al. [21], based on the intensity of immune staining and the quantity of stained cells. The intensity of staining was arbitrarily graded as absent (0), weak (1+), moderate (2+), and strong (3+). The percentage of stained cells was use d to quantify the react ion as negative (0% of positive cells), 1+ (<10% positive cells); 2+ (10–50% of positive cells); 3+ (51–80% of positive cells); 4+ (>80% of positive cells). The final value of the analysis of each tissue sample was then expressed as an absolute value through the obtained score by multiplying the two individual scores (i.e., intensity of staining score times the percentage of stained cells score) then generates a final score ranging from − (no expression) to + (weak expression), ++ (moderate expression), or +++ (strong expression). And we identified − and + as negative for G*α*s expression and ++ and +++ as positive G*α*s expression. Examples of scoring according to staining intensity and the percentage of stained cells are shown in Figure 1.

### 2.4. Statistical Analysis

SPSS version 13.0 (SPSS, Chicago, IL, USA) was used for statistical analyses. *P* values < 0.05 were considered significant. Mann-Whitney test was used to calculate the correlation between numerical variables. 2 tests were used to evaluate differences in frequency of categorical-variable groups. Spearman's rank correlation was used to analyze the correlation between continuous variables. PSA progression-free and overall survival curves were constructed by the Kaplan-Meier method and compared using the log-rank test. To evaluate the role of prognostic variables, a series of Cox proportional hazards models were fitted to PSA progression-free and overall survival data. Since PSA was a continuous estimate, with the median PSA level for the entire cohort of patients (*n* = 347) as 34.9 ng/mL, we divided the cohort into those with PSA levels ≤35 ng/mL and >35 ng/mL. The following parameters were included: PSA levels (≤35 ng/mL, >35 ng/mL); extraprostatic extension (Yes, No); involvement of surgical margins (No, Yes); involvement of seminal vesicles (No, Yes); involvement of pelvic nodes (N0, N+); Gleason scores (2–6, 7, 8–10).

## 3. Results

### 3.1. Histopathologic and Clinical Information

The median Gleason score of all patients was 7 (range: 2–10). 145 patients (41.8%) presented Gleason score of 2–6, 127 (36.6%) patients presented Gleason score of 7, and the remaining 75 cases (21.6%) presented Gleason score between 8 and 10. 49 patients (11.5%) presented TNM stage I; 125 (36.0%) patients presented stage II; 117 (33.7%) presented stage III; and 56 (16.1%) patients presented TNM stage IV. PSA progression was observed in 229 (66.0%) patients at a median interval of 123.5 month (range 7–167). Other clinicopathological features are summarized in Table 2. Moreover, prostate cancer patients who had higher Gleason scores (*P* < 0.001 and *P* < 0.001, resp.), higher TNM stages (*P* < 0.001 and *P* < 0.001, resp.), higher preoperative PSA level (*P* < 0.001 and *P* < 0.001, resp.), positive surgical margin (*P* = 0.009 and *P* < 0.001, resp.), angiolymphatic invasion (*P* = 0.004 and *P* = 0.032, resp.), extraprostatic extension (*P* = 0.031 and *P* < 0.001, resp.), and seminal vesicle invasion (*P* = 0.046 and *P* = 0.007, resp.) present shorter overall survival and PSA progression-free survival (Tables 5 and 6). PSA progression and overall survival time correlated with TNM stage, Gleason score, extraprostatic extension, positive surgical margins, and seminal vesicle invasion demonstrate the representability of study group. The number of patients with positive lymph node involvement (*N* = 34) was too small to find any significant correlation with PSA progression-free survival and overall survival.

### 3.2. Expression of G*α*s in Human Prostate Cancer

To determine the prevalence and clinical significance of G*α*s in prostate cancer tissues, we determined the expression of G*α*s protein by immunohistochemistry in a retrospective cohort of 347 tumor tissue samples from prostate cancer patients and 67 samples from patients who were diagnosed with benign prostatic hyperplasia (BPH) after tumor resection. Among the 347 patients, 114 patients had not expression of G*α*s (−); 73 patients were weak expression (+); 86 patients were moderate expression (++), and 74 patients were strong expression (+++) (as shown in Figure 1). Thus, as we described in the methods section, there were 160 (46.1%) samples positive for G*α*s expression and 187 (53.9%) samples negative for G*α*s expression in our PCa cohort. We also found that positive ratio of G*α*s expression was downregulated in metastatic PCa compared to localized PCa and BPH (*P* = 0.012 and *P* < 0.001, respectively, as shown in Table 1). In patients with BPH, there were 42 (62.7%) samples positive for G*α*s expression and 25 (37.3%) samples negative for G*α*s expression. In the patients with localized PCa, there were 151 (51.2%) samples positive for G*α*s expression and 140 (48.1%) samples negative for G*α*s expression. Whereas in the patients with metastatic PCa, there were 9 (16.1%) samples positive for G*α*s expression and 47 (83.9%) samples negative for G*α*s expression. G*α*s antibody mainly showed positive nuclear and cytoplasmic staining in prostate cancer cells. IHC staining for G*α*s was sharp and reliable, no background or nonspecific staining was observed.

### 3.3. Correlation of G*α*s with Histopathologic and Clinical Information

Then, we further evaluated the relationship between G*α*s expression and clinical features of prostate cancer patients by Pearson chi-square test or Fisher's exact test. We found that the positive ratio of G*α*s expression was decreased as the level of Gleason score or preoperative PSA increased. These result showed that there was inverse correlations between G*α*s expression and preoperative PSA and Gleason score and TNM stage at diagnosis (*P* = 0.030, *P* < 0.001, and *P* < 0.001, respectively, Table 2). But we found no specific correlation between G*α*s expression and the rest of pathological parameters (age; angiolymphatic invasion; extraprostatic extension; positive margin; seminal vesicle invasion; positive lymph node) that we evaluate in the present analysis. In the localized PCa specimens, the expression of G*α*s was correlated with preoperative PSA level, Gleason score, and TNM stage (*P* = 0.028, *P* = 0.016, and *P* = 0.011, respectively, Table 3). However, in metastatic PCa specimens, the expression of G*α*s was only associated with preoperative PSA level and Gleason score much more significantly (*P* < 0.001, and *P* < 0.001, resp., Table 4).

### 3.4. Correlation of G*α*s Expression with PSA-Free and Overall Survival

**Figure 2:**
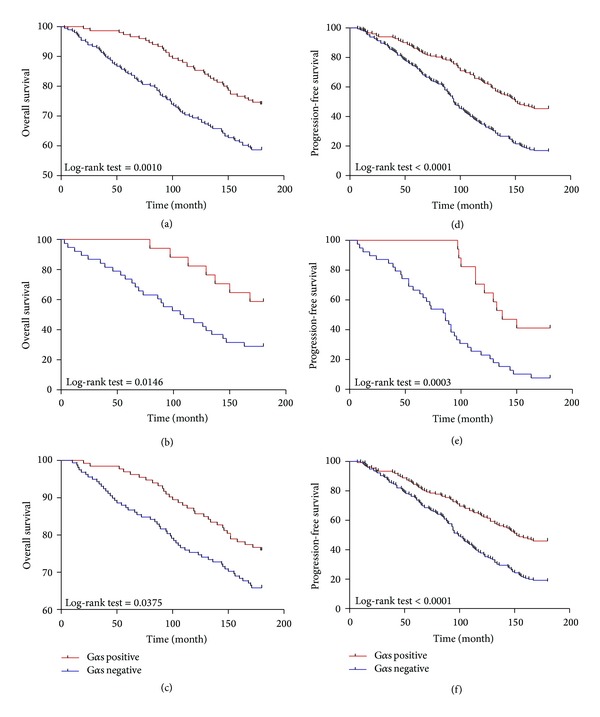
Kaplan-Meier analysis of overall survival (cumulative survival) and PSA progression-free survival of PCa patients relative to G*α*s expression. (a) Correlation of G*α*s expression with overall survival; (b) in metastatic PCa specimens, the correlation of G*α*s expression with overall survival; (c) in localized PCa specimens, the correlation of G*α*s expression with overall survival; (d) correlation of G*α*s expression with PSA-free survival; (e) in metastatic PCa specimens, the correlation of G*α*s expression with PSA-free survival; (f) in localized PCa specimens, the correlation of G*α*s expression with PSA-free survival. A statistically significant difference is shown in overall survival and PSA progression-free survival outcome between the different groups of patients, with those having positive expression of G*α*s having the better overall survival and PSA progression-free survival.

In our retrospective cohort with 347 patients, we got the detailed follow-up information of 15 years. At the time of our analysis, 121 patients died and 256 patients progressed. Median time to PSA progression for the whole cohort was 123.5 months (range 7–167 months), while the median time to death was 123.5 months (range 3–179 months). We found that patients with negative expression of G*α*s had a higher ratio of PSA progression than those with positive G*α*s expression (Table 2). More importantly, negative G*α*s expression was associated with PSA progression-free survival and overall survival. The group of patients with negative expression of G*α*s showed significantly shorter overall survival than patients with positive expression of G*α*s (*P* = 0.001, Figure 2(a)). These patients also showed a trend for shorter PSA-free survival time (*P* < 0.001, Figure 2(d)). In localized PCa specimens, negative G*α*s expression was also associated with better PSA progression-free and overall survival rate (*P* < 0.001, Figure 2(c); *P* < 0.001, Figure 2(f)). In metastatic PCa specimens, a similar trend was found between negative G*α*s expression and PSA progression-free/overall survival time (*P* = 0.0003, Figure 2(e); *P* = 0.0146, Figure 2(b)).

As expected, at the univariate level, Gleason scores, TNM stages, preoperative PSA, positive margin, angiolymphatic invasion, extraprostatic extension, and seminal vesicle invasion were associated with PSA progression-free and overall survival. Negative expression of G*α*s protein was a prognostic predictor of PSA progression-free and overall survival in PCa patients at univariate level (Tables 5 and 6).

We further conducted a multivariate Cox regression analysis to assess whether G*α*s was a prognostic predictor of survival independent of age, Gleason scores, TNM stages, preoperative PSA, positive margin, angiolymphatic invasion, extraprostatic extension, seminal vesicle invasion, and positive lymph node. Multivariate analysis showed that negative G*α*s expression was a strong independent predictor of outcome providing survival information (both PSA progression-free or overall survival) above other independent prognostic features (TNM stage, Gleason score), with a hazard ratio of 4.328 and 3.904 and a 95% confidence interval of 1.876–8.432, *P* < 0.001 (negative G*α*s group versus positive G*α*s group) and 1.278–5.873, *P* < 0.001 (negative G*α*s group versus positive G*α*s group). In our cohorts, PSA, positive margin, angiolymphatic invasion, extraprostatic extension, and seminal vesicle invasion were not independently associated with outcome at the multivariable level.

## 4. Discussion

It has been reported that the expression of G*α*s correlated inversely with serum prostate specific antigen in patients with prostate cancer and the expression of G*α*s decreased 30% to 40% after neoplastic transformation [20]. But there was no study concerning the role of G*α*s protein in the prognosis of prostate cancer patients. In the present study, we characterized the expression pattern of G*α*s protein in a large number of tissues derived from prostate cancer patients, consisting of localized and metastatic PCa, and assessed the utility of G*α*s as a prognostic marker in these patients. In agreement with previous reports, we confirmed that G*α*s expression was localized in nuclear and cytoplasm in neoplastic cells. Moreover, we found that expression of G*α*s was downregulated in metastatic PCa compared to localized PCa and BPH. And G*α*s was inversely associated with PSA level and Gleason scores both in localized and metastatic PCa. At the univariate level, G*α*s, Gleason scores, TNM stages, preoperative PSA level, positive margin, angiolymphatic invasion, extraprostatic extension, and seminal vesicle invasion were all significantly associated with PSA progression-free and overall survival. But in multivariable Cox regression analysis, only high G*α*s expression and Gleason scores were independent predictors of both PSA progression-free and overall survival. These findings support the potential clinical utility of incorporating G*α*s into clinical nomograms to help determine the risk of PSA progression and death.

The prognostic significance of G*α*s as biomarker for prostate cancer is likely to its biological functions. G*α*s is a member of GTP-binding protein superfamiliy and could independently regulate a variety of effectors including adenylate cyclases, phospholipase C*β*, and ion channels [22, 23]. However, G*α*s and T393C polymorphism in the gene GNAS1 which is encoded by G*α*s plays distinct roles in different cancers. G*α*s could augment cisplatin-induced apoptosis of lung cancer cells [10], but it inhibited cisplatin-induced apoptosis in cervical cancer or intrahepatic cholangiocarcinoma (ICC) [12]. T393C polymorphism in the gene GNAS1 was significantly associated with favorable clinical outcome of patients suffering from bladder cancer, chronic lymphocytic leukemia [15], and renal cell carcinoma [16], and significantly associated with unfavorable clinical outcome of patients suffering from ICC and breast cancer [13, 14]. In prostate cancer, previous study reported that low expression level of G*α*s was found in T2 stage PCa compared to high levels in normal controls [20]. More importantly, the expression of G*α*s was found downregulated in hormone refractory C4-2B and PC3 cell lines compared to hormone sensitive LNCaP and RWPE-1 cell lines. All these studies indicating the functionality and expression of G*α*s are selectively modified in human prostate adenocarcinoma and downregulated G*α*s levels may play an important regulatory role for cell proliferation and neoplastic transformation in prostate cancer. Thus, we did our efforts to investigate and validate whether G*α*s functioned as an efficient prognostic biomarker to predict the outcome of PCa patients. Consistent with previous studies, we found that the expression of G*α*s was much lower in metastatic PCa than it is in localized PCa. Furthermore, the patients who had the low expression of G*α*s tend to have shorter progression-free survival and overall survival time, nonetheless, metastatic or localized PCa. More importantly, multivariable Cox regression analysis proved that G*α*s was an independent predictor of prognosis in prostate cancer.

Though the answer to the discrepancy of G*α*s function in different cancers was still unclear, and the role of G*α*s in prostate cancer was also in dispute, our results were relatively easy to understand for G*α*s had a close relationship with EGFR. EGFR belongs to ErbB oncogene family which also includes ErbB-2, 3, and 4 and is comprehensively expressed in epithelial cells including prostate cancer cells. EGFR are known to regulate cell proliferation, differentiation, angiogenesis, and survival [24]. In prostate cancer, EGFR is elevated along with disease progression. It has been reported that EGFR was highly expressed in DU145 and PC3 cell lines which were hormone-independent human prostate cancer cell lines and responsive to EGF stimulation [25–27]. It was also found that prostate cancer bone metastases express significantly higher level of EGFR [28]. More importantly, EGFR expression increased as prostate cancer progressed from an androgen-dependent to an androgen-independent stage [29]. di Lorenzo et al., found 41%, 76%, and 100% EGFR expression in radical prostate ectomy hormone-sensitive and hormone-refractory metastatic patients in a cohort consisting of 76 patients with androgen-dependent and androgen-independent prostate cancer, respectively [30]. And there was a significant association between EGFR expression and higher Gleason score [31]. Many studies confirmed that overexpression of EGFR contributes significantly to the progression of prostate cancer [31–34].

Zheng et al. reported that overexpression of the stimulatory G*α*s promotes ligand-dependent degradation of epidermal growth factor (EGF) receptors and Texas Red EGF, and knock-down of G*α*s expression by RNA interference (RNAi) delays receptor degradation [35]. Recently, they demonstrated that EGF-induced, proliferative signaling occurs from EEA1 endosomes and was regulated by the G*α*s through interaction with the signal transducing protein GIV (also known as Girdin). When G*α*s or GIV was depleted, activated EGFR and its adaptors accumulate in EEA1 endosomes. Then EGFR signaling was prolonged and EGFR downregulation was delayed, which made cell proliferation greatly enhanced [36]. Basing on our finding that G*α*s was downregulated in advanced PCa and the previous studies concerning the function of EGFR in PCa, we hypothesise that downexpression of G*α*s inhibit the degradation of EGFR, then androgen receptors which are activated by EGFR were prolonged and cell proliferation increased, eventually causing tumor progression and hormone-resistant in prostate cancer. That maybe the underlying mechanism account for our results in which the expression of G*α*s was downregulated in metastatic PCa and inversely associated with PSA level and Gleason scores. But experiments would be desirable to further clarify the relationship between G*α*s and EGFR and identify their function in the progression of prostate cancer. However, such functional studies were beyond the scope of this study.

## 5. Conclusion

In summary, we discovered that G*α*s is a promising biomarker of prostate cancer patients. To our knowledge, this is the first study to describe the predictive role of G*α*s in prostate cancer. We found that the expression of G*α*s was downregulated in metastatic prostate cancer compared to localized prostate cancer. And low expression G*α*s was significantly associated with adverse clincopathological features. More important, G*α*s was an independent prognostic predictor in prostate cancer. Although our results are promising, G*α*s expression needs to be validated in relationship to outcome in the context carefully controlled clinical trials. If confirmed, application of G*α*s immunohistochemical analysis should be technically straightforward and feasible. All in all, targeting G*α*s could be a promising therapeutic strategy for enhancing the therapy effect of patients.

## Figures and Tables

**Table 1:** Comparison of G*α*s expression among different pathological categories.

Variable	Number	G*α*s positive	G*α*s negative	*χ* ^2^	*P*
All patients	347	160	187		
Metastatic PCa	56	17	39		
Localized PCa	291	143	148	6.67	0.012*
BPH	67	42	25	12.77	<0.001**

*Significant differences of G*α*s expression in metastatic PCa compared to localized PCa.

**Significant differences of G*α*s expression in metastatic PCa compared to BPH.

**Table 2:** Characterization of the cohort of 347 prostate cancer samples.

Variable	Number	G*α*s positive	G*α*s negative	*χ* ^2^	*P*
All patients	347	160	187		

Age at diagnosis (years)					
≤73	211	96	115	0.081	0.826
>74	136	64	72		
Clinical stage at diagnosis					
I	49	31	18	9.358	<0.001*
II	125	61	64		
III	117	51	66		
IV	56	17	39		
Gleason score at diagnosis					
2–6	145	83	62	15.692	<0.001*
7	127	54	73		
8–10	75	23	52		
Preoperative PSA (ng/mL)					
<35	184	99	85	9.334	0.003*
≥35	163	61	102		
Angiolymphatic invasion					
Presence	114	48	66	1.096	0.350
Absence	233	112	121		
Extraprostatic extension					
Presence	105	53	52	0.993	0.351
Absence	242	135	107		
Positive surgical margin					
Presence	128	60	68	0.048	0.911
Absence	219	100	119		
Seminal vesicle invasion					
Presence	172	78	94	0.079	0.830
Absence	175	82	93		
Positive lymph node					
Presence	34	14	20	0.369	0.590
Absence	313	146	167		
PSA progression					
Presence	213	89	124	4.153	0.047*
Absence	134	71	63		

*Significant differences of G*α*s expression among different clinical factors groups in prostate cancer samples.

**Table 3:** Characterization of the cohort of 291 localized prostate cancer samples.

Variable	Number	G*α*s positive	G*α*s negative	*χ* ^2^	*P*
Clinical stage at diagnosis					
I	49	31	18	8.972	0.011*
II	125	61	64		
III	117	51	66		
Gleason score at diagnosis					
2–6	141	81	60	8.292	0.016*
7	112	44	68		
8–10	38	18	20		
Preoperative PSA (ng/mL)					
<35	160	86	74	5.336	0.028*
≥35	131	57	84		

*Significant differences of G*α*s expression among different clinical factors groups in localized prostate cancer samples.

**Table 4:** Characterization of the cohort of 56 metastatic prostate cancer samples.

Variable	Number	G*α*s positive	G*α*s negative	*χ* ^2^	*P*
Gleason score at diagnosis					
2–6	4	2	2	15.049	<0.001*
7	15	10	5		
8–10	37	5	32		
Preoperative PSA (ng/mL)					
<35	24	13	11	11.262	<0.001*
≥35	32	4	28		

*Significant differences of G*α*s expression among different clinical factors groups in metastatic prostate cancer samples.

**Table 5:** Univariate and Multivariate analysis of clinical factors in relation to overall survival.

	Univariate HR (95% CI)	*P*	Multivariate HR (95% CI)	*P*
Negative G*α*s	5.832 (3.232–10.763)	<0.001*	3.904 (1.278–5.873)	<0.001*
Age at diagnosis	1.098 (0.921–1.284)	0.495	1.328 (0.493–4.187)	0.276
Clinical stage at diagnosis	2.287 (1.639–3.121)	<0.001*	0.723 (0.298–1.114)	0.294
Gleason score at diagnosis	3.809 (2.778–5.132)	<0.001*	2.153 (1.471–9.357)	0.004*
Preoperative PSA	2.673 (2.007–3.297)	<0.001*	2.158 (0.622–3.192)	0.429
Angiolymphatic invasion	1.224 (1.098–1.989)	0.004*	1.472 (0.897–1.677)	0.172
Extraprostatic extension	1.327 (1.211–2.019)	0.031*	0.819 (0.531–1.396)	0.491
Positive margin	2.127 (1.271–4.918)	0.009*	1.211 (0.682–2.198)	0.514
Seminal vesicle invasion	1.778 (1.281–3.711)	0.046*	1.397 (0.723–2.187)	0.283
Positive lymph node	1.698 (0.831–3.781)	0.064	0.931 (0.871–2.011)	0.592

CI: confidence interval; HR: hazard ratio; rec: recurrence.

*Significant relationships of clinical factors with overall survival.

**Table 6:** Univariate and Multivariate analysis of clinical factors in relation to PSA progression-free survival.

	Univariate HR (95%)	*P*	Multivariate HR (95%)	*P*
Negative G*α*s	5.269 (1.187–7.589)	<0.001*	4.328 (1.876–8.432)	<0.001*
Age at diagnosis	1.132 (0.809–1.727)	0.413	0.743 (0.239–3.158)	0.712
Clinical stage at diagnosis	2.787 (1.131–4.238)	<0.001*	2.135 (0.897–5.328)	0.117
Gleason score at diagnosis	5.821 (3.496–10.825)	<0.001*	3.219 (1.276–8.557)	<0.001
Preoperative PSA	1.784 (1.389–3.476)	<0.001*	0.976 (0.597–2.911)	0.061
Angiolymphatic invasion	1.829 (1.142–2.109)	0.032*	0.734 (0.549–1.291)	0.125
Extraprostatic extension	2.352 (1.399–4.569)	<0.001*	1.892 (0.897–3.219)	0.071
Positive margin	3.404 (1.778–6.091)	<0.001*	2.199 (0.782–3.988)	0.084
Seminal vesicle invasion	3.891 (1.584–5.822)	0.007*	1.329 (0.806–1.986)	0.322
Positive lymph node	2.012 (0.904–4.584)	0.091	1.212 (0.814–1.507)	0.532

CI: confidence interval; HR: hazard ratio; PSA: Prostate-specific antigen.

*Significant relationships of clinical factors with PSA progression-free survival.
